# Modeling the Longitudinal Effects of Insight on Depression, Quality of Life and Suicidality in Schizophrenia Spectrum Disorders: Results from the FACE-SZ Cohort

**DOI:** 10.3390/jcm8081196

**Published:** 2019-08-10

**Authors:** Mickaël Ehrminger, Mathieu Urbach, Christine Passerieux, Bruno Aouizerate, Fabrice Berna, Anne-Lise Bohec, Delphine Capdevielle, Isabelle Chereau, Julie Clauss, Caroline Dubertret, Aurélie Esselin, Catherine Faget, Guillaume Fond, Roxana Mihaela Honciuc, Marine Jarroir, Jasmina Mallet, David Misdrahi, Baptiste Pignon, Romain Rey, Franck Schürhoff, Hanan Yazbek, Eric Brunet-Gouet, Paul Roux

**Affiliations:** 1 Fondation Fondamental, 94000 Créteil, France; 2 HandiRESP Laboratory (EA4047) Health Sciences Department Simone Veil, University of Versailles Saint-Quentin-En-Yvelines, 78180 Montigny-le-Bretonneux, France; 3 Department of Adult Psychiatry, Versailles Hospital, 78157 Le Chesnay, France; 4 Department of Adult Psychiatry, Charles Perrens Hospital, 33076 Bordeaux, France; 5 Laboratory of Nutrition and Integrative Neurobiology (UMR INRA 1286), University of Bordeaux, 33000 Bordeaux, France; 6 Department of Psychiatry, University Hospitals of Strasbourg, 67000 Strasbourg, France; 7 Cognitive Neuropsychology and Physiopathology of Schizophrenia (INSERM U1114), University of Strasbourg, 67000 Strasbourg, France; 8 Schizophrenia Expert Centre, Le Vinatier Hospital, 69500 Bron, France; 9 PSYR2 Team, Lyon Neuroscience Research Center (INSERM U1028/CNRS UMR5292), University of Lyon 1, 69000 Lyon, France; 10 Academic Department of Adult Psychiatry, Hospital La Colombière, CHU Montpellier, 34000 Montpellier, France; 11 Neuropsychiatry: Epidemiological and Clinical Research (INSERM UMR S1061), University of Montpellier, 34000 Montpellier, France; 12 Department of Psychiatry B, Clermont-Ferrand Hospital, 63000 Clermont-Ferrand, France; 13 School of medicine, University of Clermont Auvergne, 63000 Clermont-Ferrand, France; 14 Department of Psychiatry, Louis Mourier Hospital, AP-HP, 92700 Colombes, France; 15 Institute of Psychiatry and Neuroscience of Paris (INSERM UMR1266), University Paris Descartes, 75013 Paris, France; 16 School of medicine, University of Paris Diderot, Sorbonne Paris Cité, 75018 Paris, France; 17 Ste Marguerite Hospital, AP-HM, 13009 Marseille, France; 18 CEReSS - Health Service Research and Quality of Life Center (EA 3279), School of medicine - La Timone Medical Campus, Aix-Marseille University, 13005 Marseille, France; 19 La Conception Hospital, AP-HM, 13005 Marseille, France; 20 Department of Adult Psychiatry, Charles Perrens Hospital, 33076 Bordeaux, France; 21 Institute for Cognitive and Integrative Neuroscience (CNRS UMR 5287-INCIA), University of Bordeaux, 34000 Bordeaux, France; 22 Translational Psychiatry Team (INSERM U955), Mondor University Hospital, AP-HP, 94000 Créteil, France; 23 Schizophrenia Expert Center, DHU Pe-PSY, Mondor University Hospital, AP-HP, 94000 Créteil, France

**Keywords:** Schizophrenia, suicide, insight, quality of life, depression, structural equation modeling

## Abstract

Background: Up to half of the patients with schizophrenia attempt suicide during their lifetime. Better insight is associated with better functioning but also with increased suicidality. The direction of the relationship between insight and suicidality is not clear, hence we aimed to provide new elements using structural equation modeling. Methods: Insight, quality of life (QoL), depression, and suicidality were measured at baseline and at 12 months in individuals with schizophrenia spectrum disorders. The relationships between these variables were investigated by latent difference score models, controlling for chlorpromazine doses, positive and negative symptoms, and general psychopathology. Results: 738 patients were included, and 370 completed the study. Baseline levels of insight predicted changes in suicidality, whereas baseline levels of suicidality did not predict changes in insight, suggesting that better insight underlies suicidality and predicts its worsening. Our results suggest this temporal sequence: better insight → worse QoL → increased depression → increased suicidality, while insight also affects the three variables in parallel. Conclusion: Better insight predicts a worsening of QoL, depression and suicidality. These findings contribute to our global understanding of the longitudinal influence of insight on suicidality. We advocate that insight-targeted interventions should not be proposed without the monitoring of depression and suicide prevention.

## 1. Introduction

Schizophrenia is a severe and persistent psychiatric disorder, characterized by heterogeneous symptomatology and cognitive impairments. Objective measures of functioning and subjectively reported quality of life (QoL) are negatively affected in schizophrenia and most patients are unemployed, unable to maintain social relationships or to live independently [1]. Schizophrenia is associated with a 12-fold increased relative risk of suicide and a lifetime risk of 5 to 6.5% [2,3]. Up to 50% of patients with schizophrenia attempt suicide in their lifetime [4], making it a major issue for this population. 

Between 50 and 75% of the patients with schizophrenia have poor insight [5,6], i.e., difficulties in describing their mental condition and its social consequences, attributing their symptoms to their disease and acknowledging the necessity of seeking treatment [5]. Poor insight is associated with worse clinical outcomes [6]. It is, thus, common practice to try and improve patients’ insight [7]. However, improving insight may not have only positive effects. This situation is called the “insight paradox”. It has been shown that better insight may be associated with increased depression and poorer QoL in schizophrenia [8,9,10,11]. Although research addressing this question has yielded inconsistent results, mostly because of methodological discrepancies, a recent meta-analysis [12] has provided evidence supporting the existence of a longitudinal relationship between higher insight and increased depression. Higher insight also appears to be associated with suicidality (suicide attempts and ideation) [13,14,15]. Massons et al. (2017) suggested that depression may mediate the relationship between insight and suicidality [16], and others have shown an association between higher suicidality and poorer QoL [17]. By adopting an integrative perspective, Roux et al. [18] proposed a refinement of this model in a cross-sectional study of the relationships between insight, depression, QoL and suicidality using mediation analyses with structural equation modeling (SEM). They found an indirect link between insight and suicidality, fully mediated by poor QoL and increased depression; higher insight was associated with increased depression, directly, and indirectly through QoL, and depression fully mediated the relationship between QoL and suicidality (see Figure S1).

The putative causal mechanism suggested here remains fragile, however, given the divergent results from longitudinal studies. First of all, a history of suicide attempt before the first episode of schizophrenia has been associated with better insight at the time of the first psychotic episode [15], whereas insight at that time was not associated with later suicide attempts beyond the association with depression [19], suggesting that suicide attempters may be more likely to acknowledge their mental illness. Furthermore, one study interestingly reported that baseline clinical insight predicted higher suicidality during a two-year period, whereas insight improvement during the follow-up was associated with lower suicidality [20]. This apparent contradiction highlights the possibility that longitudinal models may lead to different results, depending on whether they focus on absolute values or changes over time, and underscores the need for further investigation using robust longitudinal statistical methods such as SEM. Lastly, two potential causal directions have been reported concerning the relationships between QoL and depression: a study found that depression predicted further QoL [21], another one found that that QoL predicted further depression [22]. Here, again, the need to simultaneously test the possible directions of this relationship within the same model would help in determining the causal predominance. Latent difference score (LDS) analysis [23] provides a way to address the issue of longitudinal causal ordering between variables. It is a robust method for investigating whether the value of a variable predicts further changes in other variables.

We aimed to study the direction of the relationships between insight, QoL, depression, and suicidality in patients with schizophrenia spectrum disorders using longitudinal SEM. The main hypothesis was that better insight at inclusion predicts a worsening of suicidality, but not the reverse. We also hypothesized that better insight predicts a deterioration in QoL, that worse QoL predicts a worsening of depression, and that worse depression predicts an increase in suicidality, but not the reverse.

## 2. Experimental Section

### 2.1. Study Design

This multi-center longitudinal study included patients recruited into the FondaMental Academic Centers of Expertise for Schizophrenia (FACE-SZ) cohort between March 2010 and June 2017 by a French nationwide network of 10 schizophrenia expert centers (Bordeaux, Clermont-Ferrand, Colombes, Créteil, Grenoble, Lyon, Marseille, Montpellier, Strasbourg, and Versailles). This network was set up by the FondaMental Foundation (www.fondation-fondamental.org) and funded by the French Ministries of Research and Health to build links between systematic clinical assessment and research.

### 2.2. Participants

Schizophrenia, schizoaffective disorder, or schizophreniform disorders were diagnosed based on the Structured Clinical Interview for assessing DSM-IV-R criteria. Patients were interviewed by senior psychiatrists or psychologists specialized in schizophrenia, who were all members of the specialized multidisciplinary teams of the expert centers. We included only clinically stable patients (no admission or treatment change in the past four weeks), between 15 and 65 years of age.

The assessment protocol was conducted in accordance with the Declaration of Helsinki and was approved by the ethics review board (CPP-Ile de France IX, 18 January 2010), which required that all patients be provided with an informational letter but waived the requirement for written informed consent. However, we sought the verbal agreement of every patient before inclusion.

### 2.3. Measures

Patients were evaluated at inclusion, and one year later. 

Insight was assessed using both self-report (Birchwood Insight Scale, BIS [24,25]) and clinician-rated (Scale to assess Unawareness of Mental Disorder, SUMD [26,27]) scales, as recommended [28]. Higher BIS scores and lower SUMD scores indicate better insight. We used the mean of the first three items of the SUMD, which are general items concerning the disease (consciousness of the disease, of its consequences and of the necessity to seek treatment). The item “lack of judgement and insight” (G12) of the general psychopathology part of the Positive and Negative Syndrome Scale (PANSS) was used as a third measure of insight.

QoL was assessed using the Schizophrenia Quality of Life Questionnaire (S-QoL 18) [29], a self-report scale, explained by an eight-factor structure: psychological well-being, physical well-being, self-esteem, family relationships, relationships with friends, resilience, autonomy, and sentimental life. Higher scores indicate better QoL.

Depressive symptoms were evaluated with the Calgary Depression Rating Scale (CDS) for Schizophrenia, a structured interview scale. Higher scores indicate worse depression [30,31]. We subtracted the item “suicide” from the CDS score to avoid overlap between variables, as we used another measure for suicidality, described below.

The risk of suicide was assessed during a clinician interview that explored the patient’s experience during the past 12 months. A six-level ordinal scale ranging from 0 to 5 was used: 0, no death thoughts, suicidal ideation, or suicidal behavior; 1, patient believes life is not worth living; 2, patient has death wishes; 3, patient has already thought about committing suicide, but knowing she/he would never act; 4, patient has seriously thought about, or made plans for, committing suicide; 5, patient has attempted suicide. The scoring procedure was derived from the Columbia Suicide Severity Rating Scale [32], which proposes to report the most severe suicidal ideation category rated on a 5-point ordinal scale

The severity of schizophrenic symptoms was assessed using the Positive and Negative Syndrome Scale (PANSS) [33]. The positive symptoms, negative symptoms and general psychopathology scores were used. We subtracted the item G12 from the general psychopathology score, as this item was used as a measure of insight.

### 2.4. Analyses

We calculated Pearson’s zero-order correlations between the variables of interest. We analyzed the evolution of the variables between inclusion and follow-up using Student’s *t* tests for continuous variables (and additional Wilcoxon’s signed ranks test when non-normality was found in a variable) and Chi² tests for categorical variables. Effect sizes were estimated using Cohen’s *d* with 0.2, 0.5, and 0.8 as lower bounds for small, moderate and high effect size [34].

We compared scores at inclusion between individuals who dropped-out during the follow-up (non-completers) and those who completed both evaluations (completers) to check for potential attrition bias.

#### 2.4.1. Models

We performed structural equation modeling (SEM) using the lavaan [35] package in R. Missing data were handled with full information maximum likelihood [36]. We used LDS modeling to analyze the data [23] with a robust maximum likelihood estimator with Satorra–Bentler adjustment to account for non-normality in variables [37]. LDS models allow for testing the effect of a variable on subsequent change in another variable (Δ) to infer a direction in the association between two variables. This method is a powerful means to estimate bivariate and multivariate coupling [38]. 

We estimated the required sample size to 286 patients (see Supplementary Methods). Consensual fit indices were inspected [39,40,41]: the comparative fit index (CFI) and Tucker–Lewis index (TLI) should be > 0.9, the root mean square error of approximation (RMSEA), reported with the p-close, which should be > 0.05 and the standardized root mean residual (SRMR) should be < 0.08 to assume a good fit.

#### 2.4.2. Latent Variables and Longitudinal Invariance

Insight was defined as a latent variable with three indicators: BIS total score, mean of the three general items of the SUMD, and PANSS item G12. QoL was defined as a latent construct with eight indicators, corresponding to the dimensions assessed by the S-QoL 18. We checked for longitudinal invariance of the latent constructs (see Supplementary Methods).

### 2.5. Procedure

**Figure 1:**
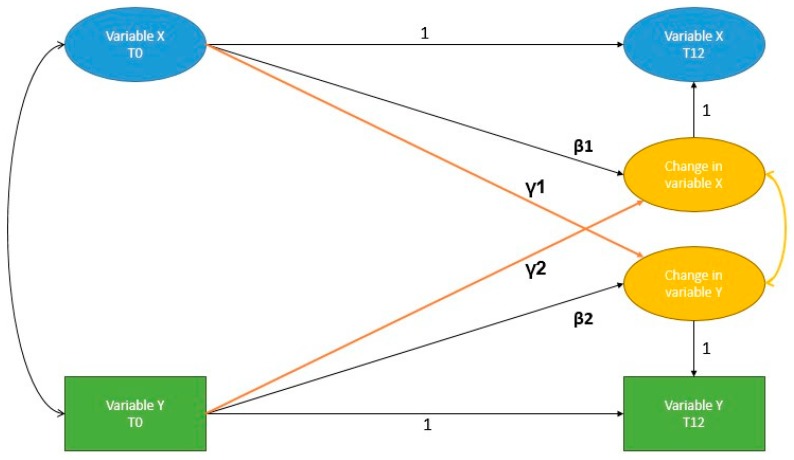
Specification of a bivariate latent difference score model (β: autoregressive path; γ: coupling path; 1: path coefficient fixed to 1)

We successively tested several models to disentangle the relationships between insight, QoL, depression, and suicidality in schizophrenia spectrum disorder. Figure 1 presents the specification of a LDS model with one latent variable X and one observed variable Y [38]; this example represents the “reciprocal” model described below. In this method, the unstandardized path coefficient from the X_T0_ and X_T12_ is fixed to 1, as is the factor loading of X_T12_ on the latent variable, representing the change in X. Paths of interest are β (autoregressive path: association between a variable’s initial value and its own change) and γ (coupling path: association between a variable’s initial value and the other variable’s change). Significant coupling paths are generally interpreted as causal paths from one variable to another. Covariance between latent changes and between variables at T0 was estimated. Indicators in latent variables at T0 were allowed to correlate with themselves at T12.

We first estimated six bivariate models obtained by combining the four variables of interest, two-by-two, and successively compared different models to test the nature and direction of putative relationships:

- **Autoregressive model**: only autoregressive paths (β)
- **Expected model**: autoregressive model + path X_T0_ → ΔY (β + γ1)
- **Reverse model**: autoregressive model + path Y_T0_ → ΔX (β + γ2)
- **Reciprocal model**: expected + reverse model (β + γ1 and γ2)

The models were compared using Chi². First, we compared the expected, reverse and reciprocal models to the autoregressive model to retain those which fit the data significantly better than the autoregressive model. We then discarded the retained reciprocal model if it did not fit the data better than the retained unidirectional model. If the reciprocal model was retained, we specified a constrained reciprocal model by fixing the unstandardized γ1 and γ2 coefficients to equality to test whether both paths were significantly different (i.e., whether a variable had a causal predominance). The direction of the “expected” path was based on theoretical assumptions. Because antipsychotics affect insight [42], QoL [43,44], depression [45], and suicide [46], chlorpromazine equivalent doses were added as a covariate. Negative, positive and general schizophrenic symptomatology were added as covariates to control for their potential confounding effect in the relationships between insight, QoL, depression, and suicide [12,47,48]. 

Finally, we estimated a multivariate model that included all the variables and simultaneously tested all the relationships contained in the retained bivariate models. 

## 3. Results

### 3.1. Participants and Evolution of the Measures

We included 738 patients between March 2010 and June 2017. A total of 370 patients participated in both evaluations, whereas 368 (49.9%) dropped out after the first evaluation. There was no significant difference between completers and non-completers, reflecting minimal attrition bias (Table S2). Only the BIS score (Student’s *t* test: *p* = 0.052, *d* = 0.15; Wilcoxon’s signed rank test: *p* = 0.087) and chlorpromazine equivalent doses (Student’s *t* test: *p* = 0.055, *d* = 0.15; Wilcoxon’s signed ranks test: *p* = 0.006) were marginally higher in the non-completers than completers, with very small effect size (<0.2).

The final sample included 344 patients after excluding those > 30% missing data. Characteristics of the sample and evolution of the variables are described in Table 1. 

We found significant changes over time in all the variables of interest with very small to small effect sizes (Table 1). Using Wilcoxon’s signed ranks test to account for non-normality in the distributions of chlorpromazine equivalent doses, the evolution of this variable over time was not significant (*p* = 0.086).

A description of diagnosis subgroups (schizophrenia and schizo-affective disorder) can be found in Supplementary Table S1.

### 3.2. Model Comparisons

#### 3.2.1. Bivariate Models

Table 2 presents the path coefficients for the retained bivariate models. Model comparisons are presented in Table S3. In sum, we found that in terms of:

- **Insight and quality of life,** the best-fitting model was the expected model, suggesting that insight causes change in QoL.
- **Quality of life and depression,** the best-fitting model was the expected model, suggesting that QoL causes change in depression.
- **Depression and suicidality,** the best-fitting model was the reciprocal model. We estimated whether the two paths were significantly different by testing a constrained model, with coupling path coefficients fixed to equality. The constrained model did not fit the data significantly worse than the unconstrained model. Thus, we cannot conclude whether one link was significantly different than the other.
- **Insight and depression,** the best fitting model was the expected model, suggesting that insight causes change in depression.
- **Insight and suicidality,** the best fitting model was the expected model, suggesting that insight causes change in suicidality.
- **Quality of life and suicidality,** the best fitting model was the autoregressive model (no coupling path), suggesting that QoL and suicidality do not directly affect each other.

#### 3.2.2. Final Multivariate Model

The global model had good fit indices: CFI = 0.942, TLI = 0.935, RMSEA < 0.05 (*p* = 1), SRMR = 0.055. The relationships found in the bivariate models remained significant when considering all the variables together, except the path from suicidality to change in depression (Figure 1 and Table S4). The model shows that insight predicts changes in QoL, depression, and suicidality. Moreover, QoL predicts changes in depression, and depression predicts changes in suicidality but not the reverse. The model accounted for 38.5% of the variance in the latent change of suicidality. 

**Figure 2:**
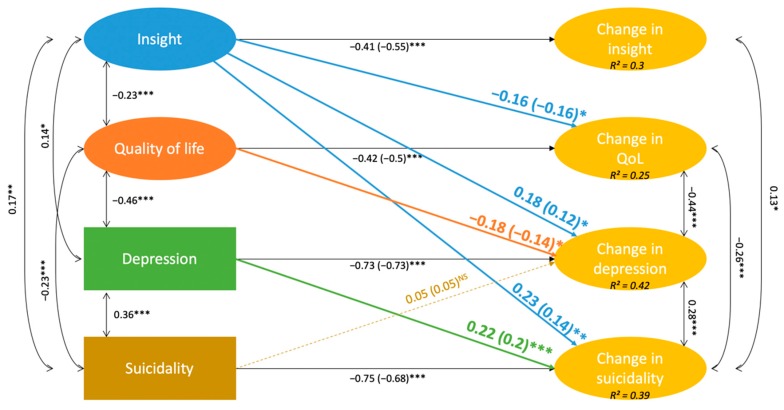
Simplified diagram of the final model. Single-headed arrows are regression paths with unstandardized (and standardized) coefficients. Double-headed arrows are covariances with standardized coefficients. For readability, only significant paths are shown. *R*^2^ is the amount of explained variance of endogenous variables. Significance levels: *p < 0.05, **p < 0.01, *** *p* < 0.001.

A simplified diagram of the global model is presented in Figure 2, presenting the estimated paths of interest. Variables at T12 are not depicted, as the paths coefficients from and to them were fixed at 1 (X_T0_ → X_T12_, ΔX → X_T12_). The zero-order correlation matrix for the variables of interest is provided in Supplementary Table S5.

The parameters of the model remained comparable after the exclusion of the three patients with schizophreniform disorders.

## 4. Discussion

The main strength of this study is that it is the first to examine the structure of the longitudinal relationships between insight, QoL, depression and suicidality in individuals with a schizophrenia spectrum disorder. The present study benefited from a large sample of patients with stabilized schizophrenia spectrum disorders (*n* = 344) and was based on statistical methods that allow the study of the dynamics of change between several variables. As recommended, we used both self-reported and clinician-rated measures of insight, and we took care to control for the potential effects of antipsychotics and negative, positive, and general schizophrenic symptomatology. 

The data strongly support a unidirectional relationship between insight and suicidality, with good insight predicting a worsening of suicidality. Our model also reflects that good insight predicts a decrease in QoL and that low QoL predicts a worsening of depression. The results were more ambiguous concerning the relationship between depression and suicidality, as the bivariate model including reciprocal relationships explained the data better than the model with only a unidirectional relationship from depression to change in suicidality. However, in the multivariate model, the path from suicidality to change in depression was not significant anymore, whereas the path from depression to change in suicidality remained significant. This result could be explained by the fact that suicide and depression may be more closely intertwined than the other clinical dimensions included in the model, and it might not be possible to disentangle them properly in schizophrenia. 

All these relationships survived the addition of positive and negative symptoms, general psychopathology, and chlorpromazine equivalent doses as covariates and the multivariate model explained 38.5% of the variance in change in suicidality. The bivariate relationship between insight and suicidality survived in the multivariate model, suggesting a direct effect of insight on suicidality and the absence of total mediation through depression and QoL. This result appears to contradict those of previous studies that showed a complete mediation of the relationship between insight and suicidality through depression and QoL [16,18,19]. This longitudinal model suggests a temporal sequence, such as a better insight precedes a decrease in QoL, and poor QoL leads to increased depression. This temporal sequence is compatible with the defense theory of insight [7], which postulates that depression occurs after an improvement of insight because of a more accurate view of the negative impact of the disorder on QoL. This association may be mediated by high internalized stigma, hopelessness, low self-esteem, or rumination [12]. The absence of direct relationships between QoL and suicidality in both the bivariate and multivariate models contradicts the results of previous studies [17,49]. The escape theory of suicide predicts that when QoL decreases, the discrepancy between patients’ current appraisal of their situation and their expectations prompts suicide. According to this theory, the relationship between QoL and suicidality is direct, but also indirect “via negative affect, especially depression and anxiety” [50]. The results of the present study suggest that the impact of QoL on suicidality is exclusively indirect: poor QoL cannot explain an increase in suicidality in the absence of depression. Moreover, our results support the assumption that better insight is associated with negative consequences in terms of QoL, depression, and suicidality. Since better insight yields better clinical outcomes but also has adverse effects, we advocate that insight-targeted interventions, such as psychoeducation, should only be carried out with particular attention paid to depressive symptoms and subjective QoL at each step of the process. 

We also found globally small but significant improvements in insight, QoL, depression, and suicidality during the 12-months follow-up period. Although difficult to interpret, this trend could be partially explained by the follow-up in the centers of expertise which provide patients with personalized recommendations concerning disease management and treatment.

Despite its strengths, this study has several limitations. First, our sample consisted of clinically stabilized patients who were not randomly selected. Thus, the results may not be generalizable to the general population of individuals with schizophrenia spectrum disorders. However, the characteristics of the sample were in accordance with previous studies in terms of insight [24] and QoL [29], thus improving the generalizability of the results to the population with stabilized schizophrenia spectrum disorders. Nearly 50% of the included patients were lost to follow-up. No survey was proposed to the non-completers, making it impossible to investigate the reasons for dropping out. However, comparisons between completers and non-completers did not show any significant differences, suggesting minimal attrition bias. We used a non-validated tool to evaluate suicidality, and both suicidal ideation and behaviors were parts of the scale, whereas different mechanisms and risk factors might play a role [17,51]. We did not control for the presence of substance use disorders, which may have a crucial influence on QoL and suicidality [52,53], and we did not distinguish between patients with schizophrenia, schizoaffective disorder, schizophreniform disorder or patients with first-episode psychosis. A complete causal model cannot be inferred from our data, as we did not actively manipulate insight, QoL, depression, or suicidality in a randomized controlled trial. Our two-timepoints design does not allow distinguishing the change between a constant change (overall rate of change across all time points) and a proportional change (depending on the adjacent measurement occasions), nor investigation of the effects of change in one variable on subsequent changes in others. Our results should, thus, be replicated by alternative longitudinal SEM, such as dual change score models or an autoregressive latent trajectory with structured residuals, which require, however, at least four and three timepoints, respectively. 

## 5. Conclusions

According to our model, higher insight detrimentally affects QoL, depression, and suicidality, and these effects add up to those which could be interpreted as a temporal cascade from QoL to suicidality via depression. This model calls for the monitoring of adverse effects of insight-targeted interventions by combining them with preventive strategies for depression. Finally, those interventions should not be proposed to patients with major depression. 

## Figures and Tables

**Table 1:** Socio-demographic and clinical characteristics of the final sample and evolution of the variable of interest between inclusion and follow-up (BIS: Birchwood Insight Scale; SUMD: Scale to assess Unawareness of Mental Disorder; PANSS: Positive And Negative Syndrome Scale; S-QoL: Schizophrenia Quality of Life; CDS: Calgary Depression Scale; Scz-aff: schizo-affective; Scz-form: schizophreniform; n: count).

	Baseline		Follow-up		*p*	*d*	Statistics
	**Mean**	**SD**	**Mean**	**SD**			
Age (years)	32.4	9.4					
Age at onset (years)	21.6	6.4					
Total duration of hospitalization (months)	7.6	10					
Insight latent score	0	1.75	0.46	1.65	<0.001	0.27	t(343) = −5.35
BIS (0–12)	8.87	2.81	9.19	2.65	0.066	0.117	t(290) = −1.84
SUMD (0–100)	30.46	32.03	22.91	29.98	0	−0.242	t(298) = 4.84
PANSS G12 (1–7)	3.13	1.52	2.82	1.55	0	−0.201	t(329) = 3.92
Quality of life latent score	0	21.27	7.95	20.94	<0.001	0.37	t(307) = −7.85
Self-Esteem (0–100)	46.76	30.21	56.88	26.31	0	0.352	t(307) = −6.96
Resilience (0–100)	55.17	25.91	59.53	25.6	0.005	0.169	t(307) = −2.85
Autonomy (0–100)	58.73	27.81	61.64	26.02	0.029	0.108	t(307) = −2.2
Physical well-being (0–100)	45.29	27.84	51.33	26.06	0	0.223	t(307) = −4.14
Psychological well-being (0–100)	51.41	27.31	58.72	26.4	0	0.27	t(307) = −4.43
Family relationships (0–100)	69.16	25.51	71.99	23.57	0.034	0.115	t(307) = −2.13
Friends relationships (0–100)	47.18	28.64	54.45	26.31	0	0.262	t(307) = −4.27
Sentimental life (0–100)	33.85	28.75	37.77	28.66	0.018	0.136	t(307) = −2.37
CDS without suicide item (0–24)	3.91	4.02	2.76	3.22	0	−0.312	t(327) = 6.12
Calgary suicide item	0.27	0.61	0.2	0.48	0.032	−0.127	t(328) = 2.15
Risk of suicide (0–5)	1.4	1.78	0.98	1.46	0	−0.297	t(313) = 5.07
PANSS Positive (7–49)	14.87	5.28	13.09	4.7	0	−0.351	t(329) = 6.54
Negative (7–49)	20.99	7.17	18.7	7.18	0	−0.315	t(329) = 6.57
General without G12 (16–105)	35.63	9.66	32.69	9.22	0	−0.308	t(329) = 5.96
Chlorpromazine equivalent doses	517.39	590.67	585.33	682.51	0.042	0.106	t(262) = −2.04
	*n*	%	*n*	%			
Sex, male	264	76.7					
Schizophrenia/Scz-aff/Scz-form disorder	269/72/3	78.2/20.9/0.9					
Hospitalized the current year	132	38.9					
Suicide attempt over the past year, yes	26	7.6	6	1.7			

**Table 2:** Unstandardized (B) and standardized (***β***) coupling and autoregressive path (→) and covariance (←→) coefficients and statistics in the retained bivariate models (QoL: quality of life, Dep: depression, Sui: suicidality, Ins: insight, ΔX: change in variable X; CFI: comparative fit index; TLI: Tucker–Lewis index; RMSEA: root mean square error of approximation; SRMR: standardized root mean square residual).

	Path	B (β)	SE	Z	*p*
QoL and depression CFI = 0.951, TLI = 0.947, RMSEA < 0.05 (*p* = 1), SRMR = 0.05	QoL → ΔQoL	−0.39 (−0.47)	0.06	−6.99	<0.001
	Dep → ΔDep	−0.71 (−0.71)	0.06	−11.73	<0.001
	QoL → ΔDep	−0.21 (−0.17)	0.08	−2.51	0.01
	QoL ←→ Dep	−0.28 (−0.46)	0.05	−6.22	<0.001
	ΔQoL ←→ ΔDep	−0.2 (−0.45)	0.04	−5.5	<0.001
QoL and suicidality CFI = 0.952, TLI = 0.949, RMSEA < 0.05 (*p* = 1), SRMR = 0.049	QoL → ΔQoL	−0.41 (−0.49)	0.06	−7.1	<0.001
	Sui → ΔSui	−0.63 (−0.58)	0.06	−11.11	<0.001
	QoL ←→ Sui	−0.16 (−0.23)	0.05	−3.5	<0.001
	ΔQoL ←→ ΔSui	−0.14 (−0.26)	0.04	−3.58	<0.001
Depression and suicidality CFI = 0.959, TLI = 0.912, RMSEA < 0.05 (*p* = 0.23), SRMR = 0.034	Dep → ΔDep	−0.66 (−0.66)	0.06	−11.86	<0.001
	Sui → ΔSui	−0.71 (−0.66)	0.06	−11.74	<0.001
	Dep → ΔSui	0.22 (0.2)	0.05	4.15	<0.001
	Sui → ΔDep	0.1 (0.1)	0.05	2.08	0.038
	Dep ←→ Sui	0.29 (0.36)	0.05	5.61	<0.001
	ΔDep ←→ ΔSui	0.2 (0.29)	0.04	4.64	<0.001
Insight and depression CFI = 0.933, TLI = 0.91, RMSEA = 0.065, SRMR = 0.047	Ins → ΔIns	−0.39 (−0.52)	0.09	−4.4	<0.001
	Dep → ΔDep	−0.64 (−0.64)	0.05	−12.1	<0.001
	Ins → ΔDep	0.25 (0.16)	0.08	3.13	0.002
	Ins ←→ Dep	0.07 (0.14)	0.03	2.03	0.042
	ΔIns ←→ ΔDep	0 (0.01)	0.02	0.15	0.88
Insight and QoL CFI = 0.928, TLI = 0.923, RMSEA < 0.05 (*p* = 0.97), SRMR = 0.056	Ins → ΔIns	−0.4 (−0.54)	0.05	−7.4	<0.001
	QoL → ΔQoL	−0.42 (−0.5)	0.06	−7.61	<0.001
	Ins → ΔQoL	−0.14 (−0.14)	0.07	−2.08	0.038
	Ins ←→ QoL	−0.1 (−0.24)	0.03	−3.39	0.001
	ΔIns ←→ ΔQoL	−0.02 (−0.11)	0.02	−1.23	0.22
Insight and suicidality CFI = 0.95, TLI = 0.932, RMSEA < 0.05 (*p* = 0.32), SRMR = 0.045	Ins → ΔIns	−0.31 (−0.48)	0.09	−3.36	0.001
	Sui → ΔSui	−0.66 (−0.61)	0.06	−11.21	<0.001
	Ins → ΔSui	0.24 (0.15)	0.08	3.12	0.002
	Ins ←→ Sui	0.01 (0.17)	0.03	2.89	0.004
	ΔIns ←→ ΔSui	0.05 (0.15)	0.03	1.98	0.048

## Supplementary Materials

The following are available online at https://www.mdpi.com/2077-0383/8/8/1196/s1, Figure S1: Model adapted from Roux et al. (2018), Supplementary methods, Table S1: Characteristics of diagnosis subgroups at inclusion; Table S2: Comparison between completers and non-completers, Table S3: Model comparisons, Table S4: Unstandardized and standardized coupling and autoregressive path coefficients and statistics in the final multivariate model, Table S5: Zero-order correlation matrix for the variables of interest.
