# Supplementary material for: Modeling the Longitudinal Effects of Insight on Depression, Quality of Life and Suicidality in Schizophrenia Spectrum Disorders: Results from the FACE-SZ Cohort
Source: J Clin Med. 2019 Aug 10;8(8):1196. doi: 10.3390/jcm8081196 (PMC6723809; doi:10.3390/jcm8081196)
Supplement: Supplementary file 1 [file jcm-08-01196-s001.pdf]

Supplementary material

1. Figure S1. Model adapted from Roux *et al*. (2018)
2. Supplementary methods
3. Table S1. Characteristics of diagnostic subgroups.
4. Table S2. Comparison between completers and non-completers.
5. Table S3. Model comparisons.
6. Table S4. Unstandardized and standardized coupling and autoregressive path coefficients and statistics in the final multivariate model.
7. Table S5. Zero-order correlation matrix between the variables of interest

Figure S1

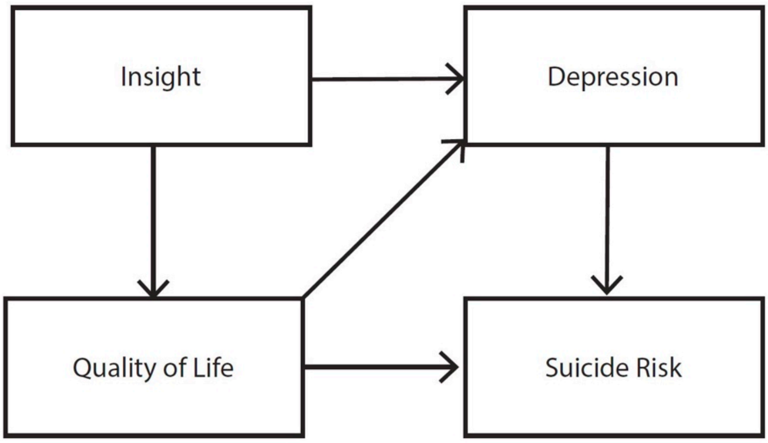

Figure 1. Model adapted from Roux *et al*. (2018)

Supplementary methods

Required sample size estimation

Velicer & Fava[1] showed that minimum sample size should not be a function of the number of indicators when the model involves latent variables, as these rules of thumb are not supported by evidence and lead to inaccurate estimates. They emphasized the importance of the number of indicators per latent variable. Marsh & Bailey[2] and Boomsma[3] suggested that the ratio of indicator per latent variable is a better basis to calculate adequate sample size. Increasing the number of indicators per latent variable maximizes the amount of information available for estimating parameters, as well as the collection of more data. Based on the previous works of Marsh[4–6], Westland[7] suggested the following formula to calculate sample size: $\mathbf{n \geq 50r^2 - 450r + 1100}$ (where r is the ratio of indicators per latent variable). We followed relatively conservative recommendations[8] and included 15 observations per variable for additional observed variables and covariates which were not included in a latent variable.

Our most complete model included eight latent variables manifested by 26 indicators; thus $r = 3.25$. According to the formula cited above, our sample should have been greater than 166 subjects. In addition to the latent variable, there were two observed variables not included in a latent variable and four covariates (positive, negative, general symptomatology, chlorpromazine equivalent dose) measured twice, thus necessitating 120 additional subjects. The total recommended sample size for our most complete model was 286 participants.

Longitudinal invariance of the latent variables

The latent variables were tested for longitudinal invariance with confirmatory factor analyses[9,10] by successively constraining parameters to be equal across times of measurement (configural invariance: constrained factorial structure; metric invariance: constrained factorial structure and factor loadings; scalar invariance: constrained factorial structure, factor loadings, and intercepts). Latent variables should reach scalar invariance to be considered equally reliable across time and be integrated in a longitudinal model.

The latent variable insight achieved scalar invariance with good fit indices: CFI = 1 and TLI = 1, RMSEA < 0.05 ($p = 0.81$), and SRMR = 0.02. The latent variable QoL achieved scalar invariance with good fit indices: CFI =

0.988 and TLI = 0.988, RMSEA < 0.05 (p = 0.98), and SRMR = 0.04. All factor loadings were significant (p < 0.001). Hence, the latent variables met the requirements to be integrated into the models.

References

1. Velicer, W.F.; Fava, J.L. Effects of variable and subject sampling on factor pattern recovery. *Psychol. Methods* **1998**, *3*, 231–251.
2. Marsh, H.W.; Bailey, M. Confirmatory Factor Analyses of Multitrait-Multimethod Data: A Comparison of Alternative Models. *Appl. Psychol. Meas.* **1991**, *15*, 47–70.
3. Boomsma, A. The robustness of LISREL against small sample sizes in factor analysis models. *Syst. Indirect Obs. Causality Struct. Predict.* **1982**, 149–173.
4. Marsh, H.W.; Balla, J.R.; McDonald, R.P. Goodness-of-fit indexes in confirmatory factor analysis: The effect of sample size. *Psychol. Bull.* **1988**, *103*, 391–410.
5. Marsh, H.W.; Hau, K.-T.; Balla, J.R.; Grayson, D. Is More Ever Too Much? The Number of Indicators per Factor in Confirmatory Factor Analysis. *Multivar. Behav. Res.* **1998**, *33*, 181–220.
6. Marsh, H.W.; Balla, J.R.; Hau, K.T. An Evaluation of Incremental Fit Indexes: A Clarification of Mathematical and Empirical Properties. In *Advanced Structural Equation Modeling Techniques : Issues and Techniques*; G.A. Marcoulides and R.E. Schumaker (eds): Mahwah, N.J, 1996; pp. 315–353.
7. Westland, C.J. Lower bounds on sample size in structural equation modeling. **2010**.
8. Stevens, J. *Applied multivariate statistics for the social sciences*; L. Erlbaum Associates: Mahwah (New Jersey), 1996; ISBN 978-0-8058-3471-0.
9. Meredith, W. Measurement invariance, factor analysis and factorial invariance. *Psychometrika* **1993**, *58*, 525–543.
10. Brown, T.A. *Confirmatory factor analysis for applied research*; Methodology in the social sciences; Second edition.; The Guilford Press: New York ; London, 2015; ISBN 978-1-4625-1779-4.

Table S1a. Characteristics of diagnosis subgroups at inclusion

	**Schizophrenia (n = 269)**		**Schizo-affective disorder (n = 72)**	
	Mean	SD	Mean	SD
**Age (years)**	32.3	9.5	32.8	9.1
**Age at onset (years)**	21.8	6.4	20.8	6.4
**Total duration of hospitalization (months)**	7.2	9.9	9.5	10.4
BIS (0-12)	8.8	2.9	9.2	2.5
SUMD (0-100)	31.2	32.3	27.9	31.7
PANSS G12 (1-7)	3.2	1.5	2.8	1.5
QOL - Self-Esteem (0-100)	47.3	30	45.4	31.4
QOL - Resilience (0-100)	56.4	24.9	50.9	29.3
QOL - Autonomy (0-100)	58.3	28.4	50.2	25.9
QOL - Physical well-being (0-100)	45.6	27.3	43.8	29.8
QOL - Psychological well-being (0-100)	51.5	27.3	51.3	28
QOL - Family relationships (0-100)	68.5	26.1	70.8	23.6
QOL - Friends relationships (0-100)	47.2	29	46.7	27.5
QOL - Sentimental life (0-100)	33.6	27.9	34.3	31.5
**CDS without suicide item (0-24)**	3.7	3.7	4.8	4.9
**Calgary suicide item**	0.3	0.6	0.3	0.6
**Risk of suicide (0-5)**	1.3	1.7	2	1.8
**PANSS Positive (7-49)**	15.1	5.4	13.8	4.9
**Negative (7-49)**	21.4	7.1	19.5	7.1
**General without G12 (16-105)**	32.9	9.3	31.1	9.2
**Chlorpromazine equivalent doses**	510	591.8	539.5	604.5
	N	%		
**Sex, male**	211	78.4	52	72.2
**Hospitalized the current year**	91	33.8	40	55.6
**Suicide attempt over the past year, yes**	17	6.3	8	11.1

Table S2. **Comparison between completers and non-completers.**

Variable	Completers (n = 370)	Non-completers (n = 368)	p	d	Statistics
**Sex (% male)**	70.9	76.8	0.086	0.063	X2 (1) =2.96
**Age (years)**	32.14 (10.11)	32.33 (9.41)	0.79	0.02	t(731.7) = -0.27
**Diagnosis (SZ/SZ-A/SZ-F)**	76.6 / 20.9 / 2.4	77.6 / 21.6 / 0.8	0.213	0.065	X2 (2) =3.1
**Age at onset (years)**	21.41 (6.61)	21.58 (6.36)	0.733	0.026	t(684.7) = -0.34
**BIS (0-12)**	8.45 (3.07)	8.89 (2.8)	0.052	0.151	t(650.7) = -1.95
**SUMD (0-100)**	34.53 (33.78)	30.48 (31.71)	0.108	-0.123	t(672.2) = 1.61
**SQOL (0-100)**	53.14 (19.03)	51.33 (18.28)	0.206	-0.097	t(679.4) = 1.27
**Calgary (0-27)**	3.92 (4.35)	4.09 (4.35)	0.605	0.039	t(703.5) = -0.52
**Risk of suicide (0-5)**	1.43 (1.74)	1.41 (1.77)	0.889	-0.011	t(700.7) = 0.14
**PANSS Positive (7-49)**	15.15 (6.38)	14.94 (5.27)	0.638	-0.035	t(675) = 0.47
**PANSS Negative (7-49)**	20.97 (7.33)	21.01 (7.19)	0.93	0.007	t(708.3) = -0.09
**PANSS General (16-112)**	36.58 (11.09)	35.54 (9.73)	0.182	-0.1	t(690.5) = 1.34
**PANSS Total (31-217)**	72.69 (21.07)	71.49 (18.09)	0.414	-0.061	t(685.6) = 0.82
**Chlorpromazine equivalent doses (mg)**	433.38 (543.95)	520.16 (596.79)	0.055	0.152	t(614.6) = -1.92

SZ: schizophrenia, SZ-A: schizo-affective disorder, SZ-F: schizophreniform disorder.

Table S3. Model comparisons. For each category, the retained model is shown in bold. Values for the AIC, BIC, Chi-square (Chisq) and Chi-square difference are rounded to the nearest decimal.

Insight and quality of life

Df	AIC	BIC	Chisq	Chisq diff	Df diff	Pr(>Chisq)	Comparison
385	23619.5	24042	633.3	-	-	-	Expected
386	23621.9	24040.5	637.6	4.3	1	0.037	Autoregressive
385	23622.3	24044.7	636	-	-	-	Reverse
386	23621.9	24040.5	637.6	1.6	1	0.203	Autoregressive
384	23620.1	24046.4	631.8	-	-	-	Reciprocal
386	23621.9	24040.5	637.6	5.8	2	0.055	Autoregressive
384	23620.1	24046.4	631.8	-	-	-	Reciprocal
385	23619.5	24042	633.3	1.5	1	0.224	Expected
384	23620.1	24046.4	631.8	-	-	-	Reciprocal
385	23622.3	24044.7	636	4.2	1	0.04	Reverse

CFI	TLI	RMSEA	RMSEA <0.05 p-value	SRMR	ECVI	Models
0.927	0.923	0.044	0.964	0.059	2.487	Autoregressive
**0.928**	**0.923**	**0.043**	**0.968**	**0.056**	**2.48**	**Expected**
0.927	0.923	0.044	0.964	0.058	2.488	Reverse
0.928	0.923	0.043	0.968	0.055	2.482	Reciprocal

Quality of life and depression

Df	AIC	BIC	Chisq	Chisq diff	Df diff	Pr(>Chisq)	Compared models
278	20730.6	21110.8	422.7	-	-	-	Expected
279	20736.7	21113.1	430.9	7.9	1	0.005	Autoregressive
278	20738.7	21118.9	430.8	-	-	-	Reverse
279	20736.7	21113.1	430.9	0.1	1	0.804	Autoregressive
277	20732.4	21116.5	422.5	-	-	-	Reciprocal
279	20736.7	21113.1	430.9	7.3	2	0.026	Autoregressive
277	20732.4	21116.5	422.5	-	-	-	Reciprocal
278	20730.6	21110.8	422.7	0.1	1	0.709	Expected
277	20732.4	21116.5	422.5	-	-	-	Reciprocal
278	20738.7	21118.9	430.8	7	1	0.008	Reverse

CFI	TLI	RMSEA	RMSEA <0.05 p-value	SRMR	ECVI	Models
0.948	0.944	0.036	0.999	0.054	1.822	Autoregressive
**0.951**	**0.947**	**0.035**	**1**	**0.05**	**1.804**	**Expected**
0.947	0.944	0.037	0.999	0.054	1.828	Reverse
0.95	0.946	0.036	1	0.05	1.81	Reciprocal

Depression and suicidality

Df	AIC	BIC	Chisq	Chisq diff	Df diff	Pr(>Chisq)	Comparison
17	9067.7	9298.1	40.4	-	-	-	Expected
18	9084	9310.6	58.6	15.8	1	0	Autoregressive
17	9078.9	9309.3	51.6	-	-	-	Reverse
18	9084	9310.6	58.6	5.6	1	0.018	Autoregressive
16	9065	9299.2	35.6	-	-	-	Reciprocal
18	9084	9310.6	58.6	20	2	0	Autoregressive
16	9065	9299.2	35.6	-	-	-	Reciprocal
17	9067.7	9298.1	40.4	4.1	1	0.042	Expected
16	9065	9299.2	35.6	-	-	-	Reciprocal
17	9078.9	9309.3	51.6	15.3	1	0	Reverse
16	9065	9299.2	35.6	-	-	-	Reciprocal
17	9065.5	9295.9	38.1	2.7	1	0.099	Constrained reciprocal

CFI	TLI	RMSEA	RMSEA <0.05 p-value	SRMR	ECVI	Models
0.916	0.841	0.081	0.014	0.042	0.513	Autoregressive
0.951	0.903	0.064	0.168	0.037	0.466	Expected
0.928	0.856	0.078	0.028	0.037	0.499	Reverse
**0.959**	**0.912**	**0.061**	**0.227**	**0.034**	**0.458**	**Reciprocal**
0.956	0.911	0.061	0.211	0.033	0.46	Constrained reciprocal

Insight and depression

Df	AIC	BIC	Chisq	Chisq diff	Df diff	Pr(>Chisq)	Comparison
69	12463.5	12782.3	169.3	-	-	-	Expected
72	12468.6	12775.8	180.3	11.3	3	0.01	Autoregressive
69	12473.2	12792	179	-	-	-	Reverse
72	12468.6	12775.8	180.3	1.4	3	0.717	Autoregressive
68	12464.6	12787.2	168.4	-	-	-	Reciprocal
72	12468.6	12775.8	180.3	11.9	4	0.018	Autoregressive
68	12464.6	12787.2	168.4	-	-	-	Reciprocal
69	12463.5	12782.3	169.3	0.8	1	0.357	Expected
68	12464.6	12787.2	168.4	-	-	-	Reciprocal
69	12473.2	12792	179	9.6	1	0.002	Reverse

CFI	TLI	RMSEA	RMSEA <0.05 p-value	SRMR	ECVI	Models
0.927	0.907	0.067	0.013	0.054	0.989	Autoregressive
**0.933**	**0.91**	**0.065**	**0.021**	**0.047**	**0.975**	**Expected**
0.926	0.901	0.068	0.007	0.053	1.003	Reverse
0.932	0.909	0.066	0.018	0.046	0.978	Reciprocal

Insight and suicidality

Df	AIC	BIC	Chisq	Chisq diff	Df diff	Pr(>Chisq)	Comparison
67	12597.7	12924.2	127.7	-	-	-	Expected
68	12603.9	12926.6	136	11.1	1	0.001	Autoregressive
67	12603.8	12930.3	133.8	-	-	-	Reverse
68	12603.9	12926.6	136	2	1	0.157	Autoregressive
66	12597.7	12928	125.7	-	-	-	Reciprocal
68	12603.9	12926.6	136	11.4	2	0.003	Autoregressive
66	12597.7	12928	125.7	-	-	-	Reciprocal
67	12597.7	12924.2	127.7	1.9	1	0.169	Expected
66	12597.7	12928	125.7	-	-	-	Reciprocal
67	12603.8	12930.3	133.8	11.1	1	0.001	Reverse

CFI	TLI	RMSEA	RMSEA <0.05 p-value	SRMR	ECVI	Models
0.945	0.925	0.056	0.208	0.052	0.884	Autoregressive
**0.95**	**0.932**	**0.054**	**0.319**	**0.045**	**0.865**	**Expected**
0.945	0.925	0.056	0.209	0.05	0.883	Reverse
0.951	0.932	0.054	0.319	0.043	0.866	Reciprocal

Quality of life and suicidality

Df	AIC	BIC	Chisq	Chisq diff	Df diff	Pr(>Chisq)	Comparison
244	19534.1	19841.4	364.1	-	-	-	Expected
245	19536.3	19839.7	368.3	3.4	1	0.067	Autoregressive
244	19537.3	19844.6	367.3	-	-	-	Reverse
245	19536.3	19839.7	368.3	1.1	1	0.286	Autoregressive
243	19535.4	19846.4	363.3	-	-	-	Reciprocal
245	19536.3	19839.7	368.3	4.6	2	0.099	Autoregressive
243	19535.4	19846.4	363.3	-	-	-	Reciprocal
244	19534.1	19841.4	364.1	0.9	1	0.351	Expected
243	19535.4	19846.4	363.3	-	-	-	Reciprocal
244	19537.3	19844.6	367.3	3.1	1	0.076	Reverse

CFI	TLI	RMSEA	RMSEA <0.05 p-value	SRMR	ECVI	Models
**0.952**	**0.949**	**0.035**	**0.999**	**0.049**	**1.53**	**Autoregressive**
0.954	0.95	0.034	1	0.047	1.524	Expected
0.952	0.949	0.035	0.999	0.049	1.533	Reverse
0.954	0.95	0.035	1	0.047	1.527	Reciprocal

Table S4. Unstandardized (B) and standardized (*β*) coupling and autoregressive path coefficients and statistics in the final multivariate model.

**Regression paths**		**B (*β*)**	**SE**	**Z**	**p**
**ΔQoL**	← QoL	-0.42 (-0.50)	0.06	-7.1	**<0.001**
	← Insight	-0.16 (-0.16)	0.07	-2.27	**0.023**
**ΔDepression**	← Depression	-0.73 (-0.73)	0.06	-11.66	**<0.001**
	← Insight	0.18 (0.12)	0.08	2.14	**0.033**
	← QoL	-0.18 (-0.14)	0.08	-2.13	**0.033**
	← Suicidality	0.05 (0.05)	0.05	1.08	0.28
**ΔSuicidality**	← Suicidality	-0.75 (-0.68)	0.06	-12.82	**<0.001**
	← Depression	0.22 (0.2)	0.05	4.06	**<0.001**
	← Insight	0.23 (0.14)	0.08	2.89	**<0.01**
**ΔInsight**	← Insight	-0.41 (-0.55)	0.07	-6.04	**<0.001**
**Covariances**					
**Insight <->**	QoL	-0.1 (-0.23)	0.03	-3.58	**<0.001**
	Depression	0.07 (0.14)	0.03	2.03	**0.043**
	Suicidality	0.09 (0.17)	0.03	2.76	**<0.01**
**QoL <->**	Depression	-0.27 (-0.46)	0.04	-6.18	**<0.001**
	Suicidality	-0.16 (-0.23)	0.05	-3.5	**<0.001**
**Depression <->**	Suicidality	0.29 (0.36)	0.05	5.63	**<0.001**
**ΔInsight <->**	ΔQoL	-0.02 (-0.09)	0.02	-1.01	0.31
	ΔDepression	0 (0)	0.02	0.03	0.97
	ΔSuicidality	0.05 (0.13)	0.02	1.98	**0.048**
**ΔQoL <->**	ΔDepression	-0.19 (-0.44)	0.04	-5.34	**<0.001**
	ΔSuicidality	-0.13 (-0.26)	0.04	-3.52	**<0.001**
**ΔDepression <->**	ΔSuicidality	0.18 (0.28)	0.04	4.53	**<0.001**

QoL: quality of life, ΔX: change in variable X.

Table S5. Zero-order correlation matrix between the variables of interest.

	At inclusion																
	BIS	SUMD	G12	Suicidality	CDS	QOL-SE	QOL-Re	QOL-Au	QOL-PhW	QOL-RFa	QOL-RFr	QOL-SL	QOL-PsW	P-Pos	P-Neg	P-Gen	Cpz eq
BIS	1																
SUMD	-0.63***	1															
PANSS-G12	-0.55***	0.73***	1														
Suicidality	0.16**	-0.17**	-0.14**	1													
CDS	0.11*	-0.05	-0.1	0.42***	1												
QOL-SE	-0.2***	0.18**	0.2***	-0.32***	-0.51***	1											
QOL-Re	-0.02	-0.03	0.05	-0.1	-0.38***	0.5***	1										
QOL-Au	-0.15**	0.08	0.07	-0.22***	-0.33***	0.49***	0.39***	1									
QOL-PhW	-0.2***	0.18**	0.2***	-0.15**	-0.38***	0.58***	0.43***	0.45***	1								
QOL-RFa	0.02	-0.05	0	0.02	-0.15**	0.14*	0.13*	0.25***	0.2***	1							
QOL-RFr	-0.07	-0.07	-0.04	-0.19***	-0.34***	0.43***	0.32***	0.34***	0.38***	0.26***	1						
QOL-SL	0.04	-0.08	0.01	-0.08	-0.25***	0.45***	0.32***	0.31***	0.36***	0.13*	0.38***	1					
QOL-PsW	-0.09	0.12*	0.19***	-0.17**	-0.38***	0.53***	0.36***	0.37***	0.48***	0.13*	0.34***	0.36***	1				
PANSS-Pos	-0.13*	0.16**	0.3***	0.06	0.13*	-0.1	0.06	-0.16**	-0.02	-0.14*	-0.02	-0.13*	-0.02	1			
PANSS-Neg	-0.16**	0.33***	0.37***	-0.04	0.19***	-0.05	-0.16**	-0.09	-0.06	-0.07	-0.25***	-0.08	-0.11*	0.26***	1		
PANSS-Gen	-0.04	0.1	0.17**	0.19***	0.52***	-0.31***	-0.23***	-0.29***	-0.3***	-0.14*	-0.29***	-0.19***	-0.3***	0.47***	0.55***	1	
Cpz eq	0.16**	-0.06	-0.09	0.12*	0.15*	-0.08	0.02	-0.04	-0.08	-0.01	0.03	0.03	-0.03	0.12*	0.05	0.15*	1
BIS_V1	0.65***	-0.51***	-0.47***	0.16**	0.02	-0.16**	-0.05	-0.13*	-0.16**	0	-0.04	0.03	-0.1	-0.18**	-0.18**	-0.03	0.11
SUMD_V1	-0.41***	0.48***	0.41***	-0.12*	-0.08	0.13*	0.03	0.02	0.13*	-0.01	-0.01	-0.06	0.07	0.15**	0.14*	0.02	-0.08
PANSS-G12_V1	-0.42***	0.49***	0.5***	-0.19***	-0.05	0.15**	0.03	0.01	0.13*	-0.05	-0.09	-0.05	0.1	0.26***	0.27***	0.13*	-0.08
Suicidality_V1	0.12*	-0.18**	-0.14*	0.39***	0.39***	-0.27***	-0.11	-0.16**	-0.23***	-0.1	-0.11	-0.1	-0.17**	-0.01	-0.07	0.12*	0.09
CDS_V1	0.22***	-0.11	-0.1	0.27***	0.49***	-0.4***	-0.22***	-0.24***	-0.34***	-0.09	-0.23***	-0.21***	-0.32***	0.04	0.06	0.25***	0.07
QOL-SE_V1	-0.17**	0.11	0.13*	-0.19***	-0.36***	0.56***	0.29***	0.41***	0.44***	0.18**	0.3***	0.32***	0.38***	-0.11*	-0.04	-0.25***	-0.06
QOL-Re_V1	-0.15*	0.15**	0.1	-0.13*	-0.31***	0.4***	0.46***	0.38***	0.44***	0.14*	0.23***	0.25***	0.3***	-0.01	-0.12*	-0.19***	-0.02
QOL-Au_V1	-0.14*	0.02	0.04	-0.16**	-0.25***	0.34***	0.24***	0.51***	0.31***	0.17**	0.24***	0.26***	0.27***	-0.16**	-0.12*	-0.23***	-0.05
QOL-PhW_V1	-0.13*	0.08	0.08	-0.23***	-0.3***	0.4***	0.3***	0.37***	0.52***	0.15**	0.28***	0.27***	0.31***	-0.05	0	-0.21***	-0.09
QOL-RFa_V1	-0.1	0.04	0.03	0.02	-0.09	0.06	0.05	0.19***	0.18**	0.55***	0.09	0.09	0.11	-0.11	-0.02	-0.05	-0.02
QOL-RFr_V1	-0.14*	0.12*	0.09	-0.14*	-0.21***	0.24***	0.23***	0.28***	0.22***	0.13*	0.47***	0.19**	0.28***	-0.07	-0.15**	-0.22***	-0.02
QOL-SL_V1	-0.04	0.08	0.03	-0.09	-0.15**	0.26***	0.17**	0.24***	0.3***	0.07	0.19***	0.44***	0.22***	-0.09	-0.12*	-0.15**	-0.08
QOL-PsW_V1	-0.09	0.13*	0.08	-0.15**	-0.32***	0.39***	0.28***	0.26***	0.34***	0.21***	0.34***	0.23***	0.44***	-0.1	-0.18**	-0.3***	-0.09
PANSS-Pos_V1	-0.07	0.06	0.19***	0.03	0.12*	-0.09	0.07	-0.12*	-0.04	-0.1	-0.07	-0.13*	-0.05	0.54***	0.14**	0.33***	0.06
PANSS-Neg_V1	-0.19***	0.23***	0.29***	-0.1	0.14*	-0.05	-0.04	-0.09	-0.03	-0.06	-0.17**	-0.1	-0.18***	0.19***	0.59***	0.36***	0
PANSS-Gen_V1	-0.05	0.04	0.16**	0.08	0.29***	-0.21***	-0.07	-0.2***	-0.2***	-0.08	-0.21***	-0.15**	-0.25***	0.28***	0.37***	0.54***	0.05
Cpz eq_V1	0.11	-0.01	-0.04	0.11	0.11	-0.05	0.01	-0.09	-0.08	-0.12*	0.04	-0.04	-0.02	0.23***	0.15*	0.24***	0.72***

	At inclusion																
	**BIS**	**SUMD**	**G12**	**Suicidality**	**CDS**	**QOL-SE**	**QOL-Re**	**QOL-Au**	**QOL-PhW**	**QOL-RFa**	**QOL-RFr**	**QOL-SL**	**QOL-PsW**	**P-Pos**	**P-Neg**	**P-Gen**	**Cpz eq**
	**At follow-up**																
	**BIS**	**SUMD**	**G12**	**Suicidality**	**CDS**	**QOL-SE**	**QOL-Re**	**QOL-Au**	**QOL-PhW**	**QOL-RFa**	**QOL-RFr**	**QOL-SL**	**QOL-PsW**	**P-Pos**	**P-Neg**	**P-Gen**	**Cpz eq**
**BIS_V1**	1																
**SUMD_V1**	**-0.58*** **	1															
**PANSS-G12_V1**	**-0.53*** **	**0.72*** **	1														
**Suicidality_V1**	**0.14***	**-0.17** **	**-0.16** **	1													
**CDS_V1**	0.09	-0.06	-0.06	**0.47*** **	1												
**QOL-SE_V1**	-0.11	0.1	0.07	**-0.38*** **	**-0.59*** **	1											
**QOL-Re_V1**	**-0.14***	**0.14***	0.05	**-0.25*** **	**-0.38*** **	**0.57*** **	1										
**QOL-Au_V1**	-0.08	0.05	0	**-0.19*** **	**-0.36*** **	**0.57*** **	**0.52*** **	1									
**QOL-PhW_V1**	**-0.11***	0.08	0.11	**-0.28*** **	**-0.46*** **	**0.61*** **	**0.55*** **	**0.52*** **	1								
**QOL-RFa_V1**	0.03	0.01	-0.02	**-0.2*** **	**-0.21*** **	**0.22*** **	**0.16** **	**0.25*** **	**0.24*** **	1							
**QOL-RFr_V1**	-0.07	0.09	-0.02	**-0.15***	**-0.27*** **	**0.42*** **	**0.37*** **	**0.42*** **	**0.36*** **	**0.23*** **	1						
**QOL-SL_V1**	-0.06	-0.04	-0.05	**-0.25*** **	**-0.38*** **	**0.42*** **	**0.36*** **	**0.35*** **	**0.39*** **	**0.17** **	**0.41*** **	1					
**QOL-PsW_V1**	**-0.13***	**0.15***	0.09	**-0.3*** **	**-0.49*** **	**0.54*** **	**0.4*** **	**0.39*** **	**0.5*** **	**0.22*** **	**0.42*** **	**0.37*** **	1				
**PANSS-Pos_V1**	**-0.19*** **	**0.32*** **	**0.39*** **	0.07	**0.12***	-0.08	0	-0.1	-0.01	**-0.15***	**-0.13***	**-0.18** **	**-0.14***	1			
**PANSS-Neg_V1**	**-0.19** **	**0.3*** **	**0.44*** **	0.04	**0.22*** **	**-0.13***	**-0.21*** **	**-0.24*** **	-0.03	-0.06	**-0.23*** **	**-0.28*** **	**-0.28*** **	**0.35*** **	1		
**PANSS-Gen_V1**	-0.07	0.11	**0.29*** **	**0.24*** **	**0.49*** **	**-0.35*** **	**-0.22*** **	**-0.29*** **	**-0.23*** **	-0.07	**-0.29*** **	**-0.33*** **	**-0.41*** **	**0.52*** **	**0.62*** **	1	
**Cpz eq_V1**	0.08	-0.11	-0.05	0.02	0.01	-0.08	-0.04	-0.03	-0.06	-0.03	-0.01	-0.06	-0.06	**0.21*** **	0.04	**0.14***	1

BIS: Birchwood Insight Scale; SUMD: Scale to assess Unawareness of Mental Disorder; G12: PANSS item G12; CDS: Calgary Depression Scale; QOL-xx: SQOL score of SE: self-esteem, Re: resilience, Au: autonomy, PhW: physical well-being, RFa: family relationships, RFr: relations with friends, SL: sentimental life, PsW: psychological well-being; P-Pos: PANSS positive symptoms; P-Neg: PANSS negative symptoms; P-Gen: PANSS general symptomatology (without item G12); CPZ eq: chlorpromazine equivalent dose
